# Robust variable selection methods with Cox model—a selective practical benchmark study

**DOI:** 10.1093/bib/bbae508

**Published:** 2024-10-14

**Authors:** Yunwei Zhang, Samuel Muller

**Affiliations:** School of Mathematics, Statistics, Chemistry and Physics, Murdoch University, 90 South St, Murdoch WA 6150, Australia; School of Mathematical and Physical Sciences, Macquarie University, 12 Wally's Walk, Macquarie Park NSW 2109, Australia; School of Mathematics and Statistics, The University of Sydney, F07 Eastern Ave, Camperdown NSW 2050, Australia; School of Mathematical and Physical Sciences, Macquarie University, 12 Wally's Walk, Macquarie Park NSW 2109, Australia; School of Mathematics and Statistics, The University of Sydney, F07 Eastern Ave, Camperdown NSW 2050, Australia

**Keywords:** robust variable selection, Cox model, variable selection, survival analysis, penalised Cox model

## Abstract

With the advancement of biological and medical techniques, we can now obtain large amounts of high-dimensional omics data with censored survival information. This presents challenges in method development across various domains, particularly in variable selection. Given the inherently skewed distribution of the survival time outcome variable, robust variable selection methods offer potential solutions. Recently, there has been a focus on extending robust variable selection methods from linear regression models to survival models. However, despite these developments, robust methods are currently rarely used in practical applications, possibly due to a limited appreciation of their overall good performance. To address this gap, we conduct a selective review comparing the variable selection performance of twelve robust and non-robust penalised Cox models. Our study reveals the intricate relationship among covariates, survival outcomes, and modeling approaches, demonstrating how subtle variations can significantly impact the performance of methods considered. Based on our empirical research, we recommend the use of robust Cox models for variable selection in practice based on their superior performance in presence of outliers while maintaining good efficiency and accuracy when there are no outliers. This study provides valuable insights for method development and application, contributing to a better understanding of the relationship between correlated covariates and censored outcomes.

## Introduction

Variable selection is a field where statistics has a huge impact on real-life challenges, especially in health and biological sciences [1, 2]. Identified important variables, for example biomarkers, can be used to guide clinical decisions, healthcare prevention strategies, and provide insights into new drug developments [3]. As high-dimensional datasets become more extensive, particularly with the inclusion of information such as last-follow-up time, and, disease or death indicators, survival models are increasingly employed to identify pivotal variables associated with the outcome of interest [4, 5]. Given the long-tailed and inherited nature of survival time distributions, as well as the presence of outlying observations in terms of their survival time, it is crucial to consider variable selection methods that are robust under such circumstances [6].

In health, various methods for variable selection are employed depending on the available data. Regularisation is one commonly utilised approach, particularly when dealing with a large number of variables in the dataset [7]. This technique can be applied in conjunction with different survival models, including the accelerated failure time model [8] and the Cox model [8, 9]. Notably, regularisation-based variable selection approaches such as the Lasso Cox model have proven instrumental in identifying important variables [10].

However, challenges arise in high-dimensional scenarios, necessitating robust methods. In such cases, employing different loss functions alongside the models’ capability of handling censored outcomes, such as censored quantile regression [11] and functional linear models [12], becomes imperative. In Supplementary Table 1, we summarise these robust methods based on the type of robust methods employed, the type of models to which they are applied, the data and outcome types, and whether they address high or low-dimensional problems from our literature search in MathSciNet and Scopus with keywords ‘robust variable selection’ or ‘robust feature selection’ & ‘survival’ until June 2024. We see a growing interest in the use of rank-based and exponential squared loss functions in survival analysis, although they have not yet been applied to the Cox model. Another popular variable selection technique as a practical alternative for classical regularisation approaches targeting high-dimensional data, is the sure independence screening (SIS) procedure, introducing a way for variable screening in large-scale biomedical data based on the Pearson correlation [13]. Recently, there has been a notable growth in the development of this method in survival analysis such as its extension to the Cox model and incorporation with the inverse probability of censoring rate, as well as its application in high-dimensional omics data such as for breast cancer and for ovarian cancer data [14–17]. For robust variable selection methods with the Cox model, we identify five R and Bioconductor packages as summarised in Supplementary Table 2. Among these, one method is not suitable for high-dimensional data when the number of predictors exceeds the number of observations. Two of the five methods exclusively perform variable selection without modeling estimated coefficients.

In practice, the predominant approach often involves fitting multiple univariate Cox models or conducting multivariate analyses using the Lasso Cox model to identify significant variables [18]. The strengths and limitations of penalised variable selection methods for censored survival biological data remain an active area of research [19], especially in the context of robust variable selection. While there have been selective reviews of variable selection for high-dimensional data [7], as well as reviews of variable selection for survival analysis [5, 7] and pioneering reviews of robust variable selection methods with applications in bioinformatics [5, 7, 20], literature on benchmarking existing methods on simulated and real datasets for their variable selection performance remains limited.

In our pursuit to contribute to the literature of comparing and contrasting variable selection methods for high-dimensional biological and medical censored survival data, we particularly recognise the necessity of addressing the challenges posed by the long-tailed survival time distribution, naturally leading us to explore robust methods tailored to handle such complexity. Upon reviewing existing literature, we observe that robust methods tailored for high-dimensional survival data are primarily inspired by regression models, limiting their applicability to the Cox proportional hazards model—a popular choice among researchers in practice [21–23]. Additionally, we observe that the key mechanism underlying the development of robust variable selection methods for high-dimensional data lies in the effective penalisation of the loss function. These findings motivate us to perform a selective review focusing on the Cox model-based robust methods for censored survival data with the comparison of different types of penalised Cox models.

Our selective review study shows the intricate relationship among the covariates, outcomes, and modeling approaches, highlighting how subtle variations can significantly impact the performance of both robust and non-robust methods. We underscore the importance of exercising caution when performing variable selection for various real-life datasets, as there is no one-size-fits-all approach. In practice, we recommend either of the two considered robust variable selection methods—the pawph [9] or the SIS method [24]—as they exhibit remarkable performance in our empirical research across datasets with varying number of variables and with outlying survival times.

The remainder of this article is organised as follows: in the Methods section, we provide a brief introduction to robust and non-robust Cox model-based variable selection approaches for censored survival data. We then introduce our simulation study design, along with a comprehensive evaluation framework. In the Results section, we first validate the effectiveness of recently proposed robust variable selection methods for the Cox model that are designed for high-dimensional survival datasets. Next, we explore various scenarios, emphasising how slight alterations can greatly impact the performances of both robust and non-robust methods. The Conclusions section contains a summary of the findings and concluding remarks.

## Methods

### Robust and non-robust variable selection methods for the Cox regression model

#### Non-robust methods

We list in Table 1 the considered non-robust variants of the penalised Cox model. For technical details for these eight methods, each implemented in the R package hdnom [25], we refer to the referenced paper shown in Table 1. The Lasso Cox approach is known to produce biased estimates, but is efficient in sparsely selecting variables. The ElasticNet Cox model uses a hybrid of Lasso and Ridge penalties, and is known to better handle multicollinearity in the data than when using the Lasso penalty alone [26]. The MCP and the SCAD are representatives of regularisation methods that have non-convex penalties. Hyperparameters may be required for such methods, for example, the SCAD penalty requires a hyperparameter that is recommended to be set to 3.7 by study [19], which may affect the method performance. In our implementation, we apply 10-fold cross validation for each run to select the optimal tuning parameter for the corresponding penalty. Then we refit the model using the selected optimal parameter.

**Table 1:** Non-robust methods

Method	Reference
Lasso Cox model (Method #1)	Tibshirani (1997) [27]
Elastic Net Cox model (Method #2)	Wu et al. (2012) [28]
MCP Cox model (Method #3)	Breheny and Huang (2011) [19]
SCAD Cox model (Method #4)	Breheny and Huang (2011) [19]
Adaptive Lasso Cox model (Method #5)	Zhang and Lu (2007) [29]
Adaptive Elastic Net Cox model (Method #6)	Xiao et al. (2015) [30]
Snet Cox model (Method #7)	Zeng and Xie (2014) [31]
Mnet Cox model (Method #8)	Zeng and Xie (2014) [31]

#### Robust methods

In this section, we briefly introduce four robust Cox model-based variable selection methods. The penalised weighted proportional hazards (pawph) model is one recently developed method and in this study, we build on this by two further variants. These are complemented by the SIS method, which is an alternative robust approach.

Method #9–#11: pawph model and its variants. The penalised weighted proportional hazards (pawph) model, recently proposed in [9], is a robust variable selection and outlier detection approach for censored survival data. The pawph method applies a Lasso-type penalty on both the beta coefficients and the variable weights.

We extended this method by replacing the Lasso-type penalty with the MCP penalty and the SCAD penalty, which we coin pawph_mcp (Method #10) and pawph_scad (Method #11), respectively. Let $ p $ be the number of covariates, $n$ denotes the sample size and let $ \mathbf{x}_{i} = (x_{i1}, x_{i2}, \ldots , x_{ip})^{T} $ denote the covariate vector for individual $ i = 1, 2, \ldots , n $, and $ \beta = (\beta _{1}, \beta _{2}, \beta _{3}, \ldots , \beta _{p})^{T} $ is the corresponding coefficient $p$-vector. Following the notation in [9] using the Lasso-type penalty, formula (3) from [9] for the the initial pawph estimator can be written as follows:


\begin{align*} (\hat{\beta}, \hat{w})&(\lambda_{1n}, \lambda_{2n}) = \mathop{\arg\min}_{\beta \in \mathbb{R}^{p}, w \in \mathbb{R}^{n}} \left\{ -\frac{1}{n} \sum_{i=1}^{n} \delta_{i} \left[\mathbf{x}_{i}^{T} \beta + \log{(w_{i})}\vphantom{\left(\sum_{j \in R_{i}} w_{j} \exp{(\mathbf{x}_{j}^{T} \beta)}\right)} \right.\right.\\ &- \left. \log{\left(\sum_{j \in R_{i}} w_{j} \exp{(\mathbf{x}_{j}^{T} \beta)}\right)} \right] + \sum_{i=1}^{n} \lambda_{1n} \frac{\left|\log{(w_{i})}\right|}{\left|\log{(\tilde{w}_{i}))}\right|} + P_{\lambda_{2n}}(\beta)\left.\vphantom{\left(\sum_{j \in R_{i}} w_{j} \exp{(\mathbf{x}_{j}^{T} \beta)}\right)} \right\}, \end{align*}

where $ P_{\lambda _{2n}}(\beta ) = \sum _{j=1}^{p} L_{\lambda _{2n}} \left ( \| \beta _{j} \| \right )$, and $ L_{\lambda _{2n}} \left ( \| \beta _{j} \| \right ) = \lambda _{2n} \| \beta _{j} \| $, with $\| \beta _{j} \| = \frac{\left |\beta _{j}\right |}{\left |{\tilde{\beta }_{j}} \right |}$, both $\tilde{w}_{i}$ and $\tilde{\beta }_{j}$ are some initial estimators.

The pawph_mcp estimator is given by


\begin{align*} (\hat{\beta}, \hat{w})&(\lambda_{1n}, \lambda_{2n}) = \mathop{\arg\min}_{\beta \in \mathbb{R}^{p}, w \in \mathbb{R}^{n}} \left\{ -\frac{1}{n} \sum_{i=1}^{n} \delta_{i} \left[\vphantom{\left(\sum_{j \in R_{i}} w_{j} \exp{(\mathbf{x}_{j}^{T} \beta)}\right)}\mathbf{x}_{i}^{T} \beta + \log{(w_{i})}\right.\right. \\ &- \left.\log{\left(\sum_{j \in R_{i}} w_{j} \exp{(\mathbf{x}_{j}^{T} \beta)}\right)} \right] + \sum_{i=1}^{n} \lambda_{1n} \frac{\left|\log{(w_{i})}\right|}{\left|\log{(\tilde{w}_{i}))}\right|} \left. \vphantom{\left(\sum_{j \in R_{i}} w_{j} \exp{(\mathbf{x}_{j}^{T} \beta)}\right)}+ P_{\lambda_{2n,\gamma}}(\beta) \right\}, \end{align*}

where $ P_{\lambda _{2n,\gamma }}(\beta ) = \sum _{j=1}^{p} M_{\lambda _{2n,\gamma }} \left ( \| \beta _{j} \| \right )$, and


\begin{align*}& M_{\lambda_{2n},\gamma}(\| \beta_{j} \|) = \begin{cases} \lambda_{2nj} \| \beta_{j} \| - \frac{\| \beta_{j} \|^{2}}{2\gamma}, & \textrm{if}\ \| \beta_{j} \| \leq \gamma\lambda_{2nj} \\ \frac{1}{2} \gamma{\lambda_{2nj}^{2}}, & \textrm{if}\ \| \beta_{j} \|> \gamma\lambda_{2nj}, \end{cases} \end{align*}

where $\gamma $ is a tuning parameter.

The pawph_scad estimator is given by


\begin{align*}& \begin{aligned} (\hat{\beta}, \hat{w})&(\lambda_{1n}, \lambda_{2n}) = \mathop{\arg\min}_{\beta \in \mathbb{R}^{p}, w \in \mathbb{R}^{n}} \left\{ \vphantom{\left(\sum_{j \in R_{i}} w_{j} \exp{(\mathbf{x}_{j}^{T} \beta)}\right)}\right. -\frac{1}{n} \sum_{i=1}^{n} \delta_{i} \left[\vphantom{\left(\sum_{j \in R_{i}} w_{j} \exp{(\mathbf{x}_{j}^{T} \beta)}\right)}\right. \mathbf{x}_{i}^{T} \beta + \log{(w_{i})} \\ &- \left.\log{\left(\sum_{j \in R_{i}} w_{j} \exp{(\mathbf{x}_{j}^{T} \beta)}\right)} \right] + \sum_{i=1}^{n} \lambda_{1n} \frac{\left|\log{(w_{i})}\right|}{\left|\log{(\tilde{w}_{i}))}\right|} \left. \vphantom{\left(\sum_{j \in R_{i}} w_{j} \exp{(\mathbf{x}_{j}^{T} \beta)}\right)}+ P_{\lambda_{2n,\gamma}}(\beta) \right\}, \end{aligned} \end{align*}

where $ P_{\lambda _{2n,\gamma }}(\beta ) = \sum _{j=1}^{p} S_{\lambda _{2n,\gamma }} \left ( \| \beta _{j} \| \right )$, and


\begin{align*}& \begin{split} S_{\lambda_{2n},\gamma}(\| \beta_{j} \|) = \begin{cases} & \lambda_{2nj} \| \beta_{j} \|, \quad \textrm{if}\ \| \beta_{j} \| \leq \lambda_{2nj} \\[4pt] & {-\frac{\| \beta_{j} \|^{2} -2\gamma\lambda_{2nj} \| \beta_{j} \|+ \lambda_{2nj}^{2}}{2 \left(\gamma -1\right)}}, \\ & \quad \textrm{if}\ \lambda_{2nj} < \| \beta_{j} \| \leq \gamma\lambda_{2nj} \\[4pt] & \frac{\lambda_{2nj}^{2} (\gamma^{2} - 1)}{2(\gamma - 1)}, \quad \textrm{if}\ \| \beta_{j} \|> \gamma\lambda_{2nj}, \end{cases} \end{split} \end{align*}

where $\gamma $ is a tuning parameter.

Method #12. SIS Cox model.

The SIS method has been extended for the Cox model in [24]. For full technical details, which we omit here due to space constraints, we refer to [24].

### Random Survival Forest

We also include the Random Survival Forest (RSF) method [32] in our comparison. This method is a non-robust and non-parametric approach for variable selection but not for the estimation of the $\beta $ vector.

### Simulation design

We use multiple simulation scenarios to examine model variable selection performances for method #1–#12. To justify the performances of recently developed robust variable selection methods with the Cox model, we first adopt similar simulation designs based on the pawph study (Design 1) [9] and the SIS study (Design 2) [24]. Furthermore, to reveal the latent structure in the simulation design that may favor one method compared to the other, we apply a combination of the correlation structure from these two simulation designs with varying beta parameter sets (see Scenarios 1–6 for further details). We also apply the RSF method under these simulation designs.

#### Design 1

The same correlation matrix from the pawph study [9] is used in this design. For the beta parameter, case (a) in [9] with $(\beta _{1},\beta _{2},\beta _{3}) = (1, 2, -1)$ is used. We set $n=300$ and $p=30$. Therefore, $\beta _{4} = \ldots = \beta _{30} = 0.$ In survival literature, outliers typically refer to individuals whose survival time is either too long or too short [33, 34]. We follow the study by [9] and correspondingly set the outlier proportion to 5%. These outliers are created by adding a fixed constant time that is equal to the median of the true survival time to the true survival time for randomly selected individuals.

#### Design 2

The same simulation designs from the SIS study [24] are used in our Design 2, where we have Case 1–Case 6 in [24] with different correlation matrices, $\beta $ parameter sets, $n$, and $p$.

#### Scenario 1–6

We generate standardised pseudo-normal observation vectors $\mathbf{x}_{i}$ with correlation matrix


\begin{align*} & \left(\begin{array}{cccccccccc} 1 & 0.9 & 0.9 & 0 & 0.1 & 0.1 & {0.1} & \dots & {0.1} & 0.1 \\ 0.9 & 1 & 0.9 & 0 & 0.1 & 0.1 & {0.1} & \dots & {0.1} & 0.1 \\ 0.9 & 0.9 & 1 & 0 & 0.1 & 0.1 & {0.1} & \dots & {0.1} & 0.1 \\ 0 & 0 & 0 & 1 & 0.7 & 0.7 & {0.1} & \dots & {0.1} & 0.1 \\ 0.1 & 0.1 & 0.1 & 0.7 & 1 & 0.7 & {0.1} & \dots & {0.1} & 0.1 \\ 0.1 & 0.1 & 0.1 & 0.7 & 0.7 & 1 & {0.1} & \dots & {0.1} & 0.1 \\{0.1} & {0.1} & {0.1} & {0.1} & {0.1} & {0.1} & {1} & \dots & {0.1} & {0.1} \\ \vdots & \vdots & \vdots & \vdots & \vdots & \vdots & \vdots & \ddots & \vdots & \vdots \\{0.1} & {0.1} & {0.1} & {0.1} & {0.1} & {0.1} & {0.1} & \dots & 1 & {0.1} \\ 0.1 & 0.1 & 0.1 & 0.1 & 0.1 & 0.1 & {0.1} & \dots & {0.1} & 1 \end{array}\right). \end{align*}

That is, the first six variables have a correlation structure consisting of three blocks, and the remaining variables have pairwise constant correlations of 0.1 with all explanatory variables in the data. We apply $\beta $ parameter sets 1-6 as shown in Table 2 with $n=300$, $p=30$ in the low-dimensional setting and $p=1000$ in the high-dimensional setting. To compare the impact of sample size, we further simulate datasets with $n$=1000, $p$=1000 for Scenario 3. We vary the outlier proportion over 0, 0.001, 0.002, 0.005, 0.01, 0.02, 0.05, 0.1, and 0.2. We use the ‘ceiling’ function in R to obtain integer numbers of outliers according to various outlier percentages. For the censoring distribution, we choose the uniform distribution over $[0, 10]$. We consider a Weibull distribution with parameters $\alpha = 5$ and $\beta = 0.5$ for the baseline hazard.

**Table 2:** Regression coefficients in the six scenarios

Scenario	$\beta _{1}$	$\beta _{2}$	$\beta _{3}$	$\beta _{4}$	$\beta _{5}$	$\beta _{6}$	$\beta _{7},\ldots ,\beta _{p-1}$	$\beta _{p}$
1	10	-3	3	-10	-3	3	$0,\ldots ,0$	5
2	10	0	3	-10	-3	0	$0,\ldots ,0$	5
3	2	-3	3	-2	-3	3	$0,\ldots ,0$	2
4	0	0	3	0	-3	0	$0,\ldots ,0$	5
5	10	0	0	10	0	0	$0,\ldots ,0$	5
6	2	0	0	-2	0	0	$0,\ldots ,0$	2

For each simulation scenario, we run 500 repeats and perform the evaluation for each repeat.

### Evaluation

We apply a hybrid evaluation approach where we use eight metrics as used in at least one of the studies in [24, 35, 36]. Specifically, as criteria to evaluate how well a method identifies the true variables we consider the number of true positives, the number of false positives, the F1 score, the mean model size, and the estimated probability that the true model is selected. Additionally, we also consider the estimation of the effect of variables. Details of each evaluation metric is given below. We do not evaluate model prediction ability as this is not the focus of this study.

#### Identification of true variables

We choose the number of true positives (TP), the number of false positives (FP) and the F1 score to evaluate a method’s ability to identify true variables, that is, variables having corresponding non-zero regression coefficients. Mean model size, defined as the mean of the number of all selected variables, is included to get an insight of which approach tends to select a larger model and which does not. We further include the estimated probability, which reflects the probability that the true model is selected. The true model refers to the model that includes all true active variables but none of the inactive variables.

#### Efficient estimation of coefficients

To measure the difference between the estimated regression coefficients and their true values, we use the mean estimate, the mean absolute error and the mean square error. Thus, denoting the true regression coefficients as $\beta _{j}$ and corresponding estimated values as $\hat \beta _{jr}$, $j=1,\ldots ,p$, and for $r=1,\ldots ,m$ simulation runs, we have

-
$\textrm{EST} = \textrm{mean estimate}=\frac{1}{m} \sum _{r=1}^{m} \hat{\beta }_{jr}$ ;
-
$\textrm{MAE} = \textrm{mean absolute error}=\frac{1}{m} \sum _{r=1}^{m} |\hat{\beta }_{jr}- \beta _{j}| $ ;
-
$\textrm{MSE} = \textrm{mean square error}=\frac{1}{m} \sum _{r=1}^{m} (\hat{\beta }_{jr}- \beta _{j})^{2}$ .

## Results

### Validation of robust variable selection methods

**Figure 1:**
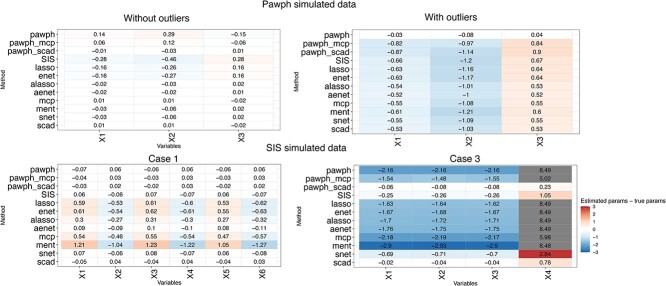
Effectiveness of recent developed robust variable selection methods with the Cox model. (1) Top panel: results from the pawph approach with left columns under the simulation setting without outliers and right columns with outliers. Rows in the heatmap are methods and columns are variables in the data. Methods are ordered by robust methods and then non-robust methods. Colors represent the difference of values, i.e. ‘the value of estimated parameters - the value of true parameters’. (2) Bottom panel: results from the SIS approach with left columns form Case 1 and right columns from Case 3. For each heatmap, rows represent methods and columns represent variables in the data. Colors represent the same as in the top panel.

We study the pawph and the SIS approach. The upper panel of Fig. 1 depicts the performance of the pawph method in a simulated setting as described in the corresponding study [9]. We observe that under the ‘with outlier’ scenario, the pawph method outperforms other approaches across all evaluation metrics (indicated by white boxes for all variables in Fig. 1), including the distance between estimates and true parameters, the mean absolute difference, and the mean squared difference. Conversely, under the scenario without outliers, both the pawph and SIS method are suboptimal here (indicated by intense colored boxes in Fig. 1). Meanwhile, penalised Cox models such as the adaptive Lasso Cox model and the MCP Cox model have superior performance (indicated by white colored boxes in Fig. 1), suggesting that advanced penalised methods may suffice for low-dimensional datasets in the absence of outliers.

The effectiveness of the SIS method is confirmed through the replication of results outlined in the study [24]. The bottom panel in Fig. 1 illustrates two cases from the SIS method study. We select Case 1 to demonstrate that both the pawph and SIS method exhibit strong performance under this setting, along with advanced penalised Cox models including the snet Cox model and the SCAD Cox models. Conversely, Case 3 presents a different scenario, wherein only the SIS method emerges as one of the top-performing methods, with the pawph approach falling short.

### Comparison of estimates

**Figure 2:**
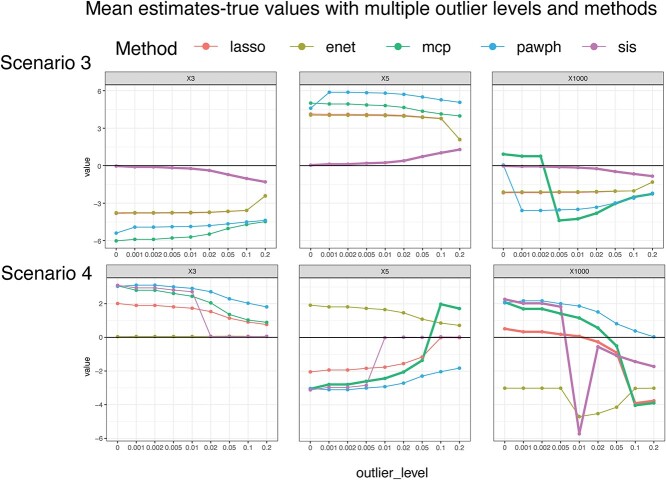
Comparison of estimated values across different outlier percentages, methods, and scenarios. Colors of lines represent different methods. Each row represents one scenario. Each plot shows results for one parameter with $x$-axis containing different outlier percentages varying from 0% (0) to 20% (0.2) and $y$-axis showing the difference with the true parameter. The horizontal $y$=0 line represents perfect estimations. Lines that either represent the best performing model or models that cross the $y$=0 line are thickened.

To gain insights into the variability of estimation performance, particularly assessing whether both the pawph and SIS method outperform baseline penalised Cox models under different scenarios with the presence of outliers, we adopt characteristics from both the simulation settings of pawph and SIS (see Method, Simulation design). By varying the parameters across Scenario 1 to 6, we find that no single approach performs optimally across all scenarios in terms of parameter estimation. Figure 2 illustrates differences among the Lasso Cox model, Elastic Net Cox model, MCP Cox model, pawph, and SIS in estimating the true parameters in dependence of the percentage of outliers in the data. As the number of outliers increases there is no monotone trend in terms of estimation bias. Fluctuations in estimates are observed across all methods as the scenarios vary. Additionally, shifts between overestimates and underestimates are evident, as indicated by lines crossing the $y$=0 horizontal line in Fig. 2.

The SIS approach consistently outperforms all other methods across all outlier percentages and variables in Scenario 3. However, for the pawph approach, we do not observe a single scenario where pawph consistently outperforms all other methods across all variables and outlier percentages. Observing that no single approach is universally superior in all scenarios, it would be advantageous to determine which method works the best under specific circumstances. However, drawing a simple rule to guide this determination proves challenging. Even slight changes in the desired important variables, parameter scales, and relationships among selected variables can lead to significant differences in the performance of various approaches.

### Comparison of variable selection

**Figure 3:**
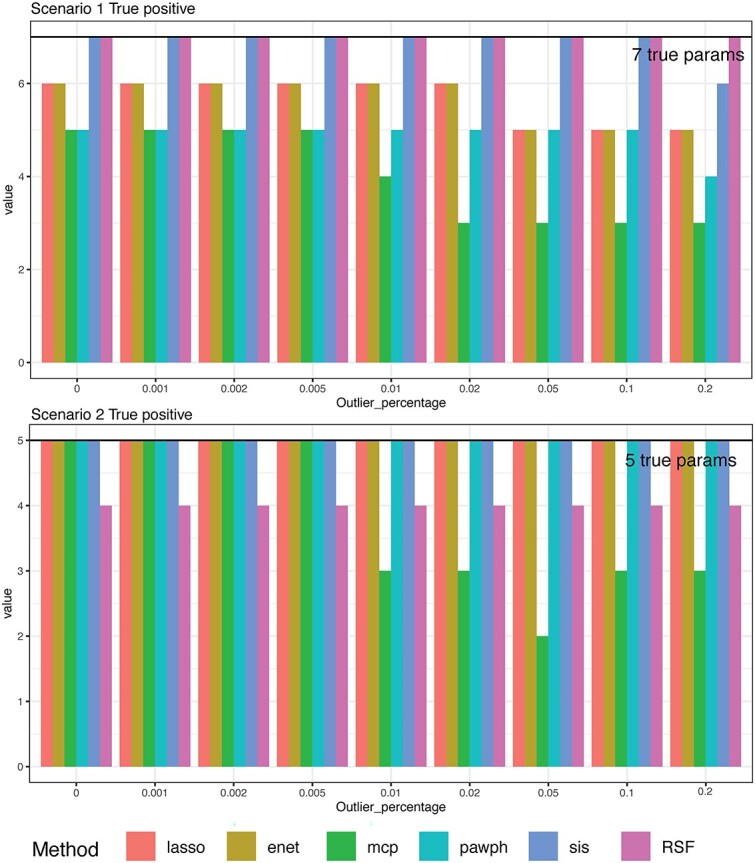
Truly identified variables. Bar plots for each scenario considered in this study with $x$-axis representing outlier percentage and $y$-axis representing the number of true non-zero parameters identified. The horizontal bold black line shows the true number of non-zero parameters.

To evaluate the effectiveness of each method in identifying important variables, we compute several metrics including the number of correctly selected variables, falsely selected variables, mean model size, and the probability of identifying the correct model (Supplementary Figs 1–3.). We visualise the number of correctly selected variables for Scenario 1 and 2 under the high-dimensional setting ($n=300$, $p=1000$) as illustrated in Fig. 3. Notably, SIS consistently identifies the true variables in all scenarios across various outlier percentages, except for Scenario 1 with an outlier percentage of 20%. The pawph method tends to select smaller models, resulting in the omission of true variables. However, for Scenario 4–6 where only three true variables are present in the design, all methods successfully identify these important variables. We observe the RSF method is able to select all correct variables under Scenario 1 and misses variables under Scenario 2. We also provide a real data variable selection comparison in the supplementary results.

### Impact of the sample size

**Figure 4:**
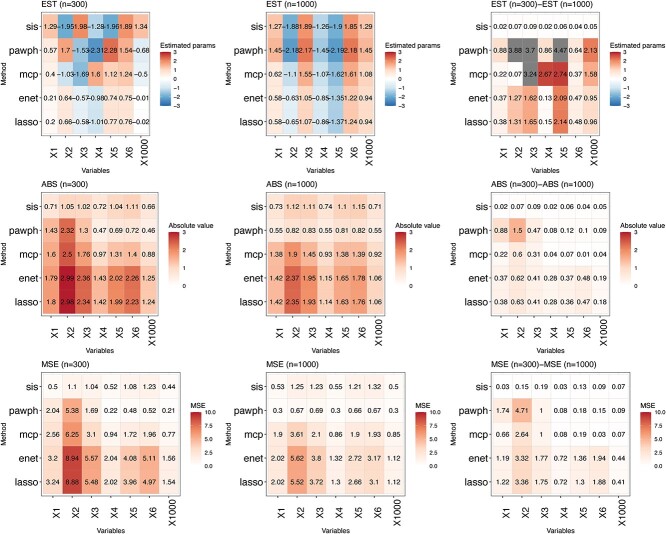
Low- and high-dimensional data comparison. The first column shows the low-dimensional results when $n=300$ and the second column shows the high-dimensional results when $n=1000$ observations. The third column shows the differences between the low- and the high-dimensional results. Colors visualise the magnitude of three evaluation metrics: mean estimates (first row), mean absolute errors (second row) and mean square errors (third row).

Another critical aspect that influences model performance in variable selection is the sample size $n$. Both studies [9] and [31] demonstrate that when increasing the dimensionality to $p=1000$, both pawph and SIS perform relatively well. To further investigate this, we compare $n=300$ with $n=1000$ in Scenario 3. We visualise the estimated parameters, absolute errors, and mean squared errors using heatmaps in Fig. 4. The last column in Fig. 4 provides the absolute differences in each criteria for the high-dimensional case. As expected, for the differences in the absolute errors (second row, third column in Fig. 4) and the mean squared errors (third row, third column in Fig. 4), the majority of these differences are small, which is visualised by the many close to white boxes. This indicates that pawph and SIS effectively handle high-dimensional datasets when $p$ is close to $n$. Additionally, comparing the absolute error results and the mean square error results between $n=300$ (first column, second and third row in Fig. 4) with $n=1000$ (second column, second and third row in Fig. 4), we observe that pawph performs better under the high-dimensional setting compared to the low-dimensional setting.

## Conclusion

Variable selection is a heavily researched area in statistics. With the rise of interdisciplinary research, its applications have expanded significantly across various fields, particularly in medicine and biology. Real-life datasets present many challenges, including how to select a good subset from a large number of variables, especially when the signal is potentially weak (i.e. the true regression coefficients are small) when modeling the data with regression-type models, and when dealing with diverse types of outcome variables.

In our study, instead of conducting a systematic review of all existing methods and delving into their technical details, we opted for a selective review focusing on recently developed robust variable selection approaches for the Cox model. Through variations in the design matrix, the coefficients of covariates, outlier percentages, and the ratio of the number of observations to the number of variables, we concluded that slight alterations can greatly impact the performance of both robust and non-robust methods. We recommend to users of statistics to consider robust Cox models for variable selection in practice based on their good overall efficiency and accuracy, especially in the presence of outliers.

Our study justifies the performance of two recently developed robust Cox models, pawph and SIS, for variable selection in high-dimensional censored survival data, providing insights for future method development by harnessing the strengths of these modern approaches. With the pawph approach working on the weighted version of the negative log partial likelihood function through the addition of new penalised terms, and the SIS approach finding its success by analysing the correlation structure, there is also potential for incorporating time-varying covariates, left-censoring, and interval-censoring in the development of robust Cox model variable selection methodologies. Both pawph and SIS are shown (in Results: validation of robust variable selection methods) to effectively handle rowwise outliers. We remark again that outliers in survival data often occur in the response information by either a long or a short survival time for an individual. There are other types of outliers, for instance, cellwise outliers [37], where methods are developed when explanatory data is corrupted. To the best of our knowledge, methods that are tailored for cellwise outliers in Cox models have yet to be designed. This is an open research area in survival analysis. Additionally, we validate the performance of the SCAD and MCP penalised Cox models, as previous review studies have confirmed their effectiveness in regression models [38].

The Cox model stands out as one of the most frequently used survival analysis methods. Comparing and contrasting different approaches provides essential guidance for applied and translational researchers. We emphasise the need for caution when performing variable selection for various real-life datasets, as there is no one-size-fits-all approach. Depending on the data of interest and domain knowledge, researchers may need to employ multiple methods before drawing a conclusion. We recommend the use of the pawph and the SIS method in practice, as they have demonstrated outstanding performance for a large number of variables with outlying survival times. The pawph approach tends to select a small set of important variables, thus reducing computational burden and enhancing time efficiency. This is particularly valuable in practical applications. Moreover, its enhanced interpretability facilitates actionable improvements in clinical decision-making. The SIS method, which successfully selected all important variables in all except one scenarios in our study, demonstrates its superior variable screening performance in high-dimensional setting. From the application perspective, the pawph method has been implemented in the software R. We stress that this R implementation of pawph depends on downloading and installing other packages. The SIS method has been implemented in R and in Python. Both packages are straightforward to use.

The performance evaluation of variable selection methods for high-dimensional censored survival data remains an open research area. It is not straightforward to conclude which penalty works best under various scenarios. Our study represents an initial step toward unravelling the complex relationship between the data and model performance, but there is much more work to be done. The simulation design we employ is limited to linear relationships among covariates. Future work could explore non-linear relationships. Additionally, our study only addresses robust methods that primarily handle outlier individuals in the data. We acknowledge the existence of other types of robust methods, such as those addressing model misspecification, which warrant further review in high-dimensional survival analysis.

**Key Points**

- A selective practical benchmark study for robust and non-robust Cox model-based variable selection methods for high-dimensional censored survival data.
- Even small variations in the data can significantly impact method performances.
- We recommend the use of robust Cox models for variable selection in practice.

## Supplementary Material

Supplymentary_file_revision_v2_bbae508

## Data Availability

Data and codes are available at: https://github.com/yunwezhang/Robust-variable-selection-with-Cox-model.

## Author descriptions

Yunwei Zhang is a Postdoctoral Fellow in the School of Mathematical and Physical Sciences at Macquarie University. Her research interests are statistical machine learning, feature selection methods, survival analysis, simulation algorithms, and graphical visualisations. Samuel Muller is a Professor and the Head of the School of Mathematical and Physical Sciences at Macquarie University. His research interests lie in discovering, designing, and building statistical models that better population and planet health.
