# Supplementary material for: Robust variable selection methods with Cox model—a selective practical benchmark study
Source: Brief Bioinform. 2024 Oct 14;25(6):bbae508. doi: 10.1093/bib/bbae508 (PMC11472364; doi:10.1093/bib/bbae508)
Supplement: Supplymentary_file_revision_v2_bbae508 [file supplymentary_file_revision_v2_bbae508.docx]

Supplementary Material

Tables:

Supplementary Table 1. Selected summary of robust variable selection methods for high dimensional survival data analysis.

| Method | Models included | Penalty type | Outcome types | High or low dimensional | Domain | Reference |
| --- | --- | --- | --- | --- | --- | --- |
| Check loss function | Linear model; censored quantile regression model | Lasso penalty | Continuous | High and low | Clinical data; riboflavin data | Wang (2022). Wang (2013). |
| Least absolute deviation loss | Linear model | Lasso penalty | Continuous | Low | Wood gravity data; concrete slump data | Jiang (2021). |
| Rank-based loss | Functional linear model; accelerated failure rate model; linear model | Lasso and adaptive Lasso penalty | Continuous  outcome; Survival outcome | High and low | Sugar spectra data; clinical data; Cancer genomic data | Su (2024).  Bindele (2022).  Xu (2010).  Cai (2009).  Johnson (2009). |
| Exponential squared loss | Linear model; spatial (varying coefficient) model; time series model; spatial autoregressive model; partially linear model | Adaptive Lasso penalty | Continuous; time series | Low | Economic data; temperature data; salinity data | Wang, X. (2023).  Wang, Y. (2023).  Zou (2023).  Wu (2022).  Song (2021).  Jiang (2019). |
| S-loss | Linear model | Adaptive Elastic Net penalty | Continuous | High | Chemical composition data | Kepplinger (2023). |
| Absolute loss; Huber’s loss; Tukey’s loss | Threshold model | Lasso penalty | Continuous | Low | Economic data | Li (2020). |
| Network based loss | Accelerated failure rate model | Modified MCP penalty | Survival outcome | High | Cancer genomic data | Ren (2019). |
| Density power divergence loss | Linear model | Adaptive Lasso | Continuous | High | Cancer genomic data | Ghosh (2024). |
| Gene environment interaction based loss | Accelerated failure rate model | MCP penalty | Survival outcome | High | Cancer genomic data | Shi (2014). |
| Modified weighted hazard function loss | Coxph model | Adaptive weighted Lasso penalty | Survival outcome | Highand low | Clinical and omics data | Luo (2022). |

Supplementary Table 2. Implementation of methods.

| Package name | Notes | Link |
| --- | --- | --- |
| pawph | n<p available and has estimated parameters returned. | <https://github.com/r08in/PAWPH> |
| SIS | n<p available and has estimated parameters returned. | <https://cran.r-project.org/web/packages/SIS/SIS.pdf> |
| rbsurv | No estimated parameters returned. | <https://www.bioconductor.org/packages/release/bioc/html/rbsurv.html> |
| coxrobust | n<p is not allowed. | <https://cran.r-project.org/web/packages/coxrobust/index.html> |
| MXM | No estimated parameters returned. | <https://cran.r-project.org/web/packages/MXM/index.html> |

Figures:


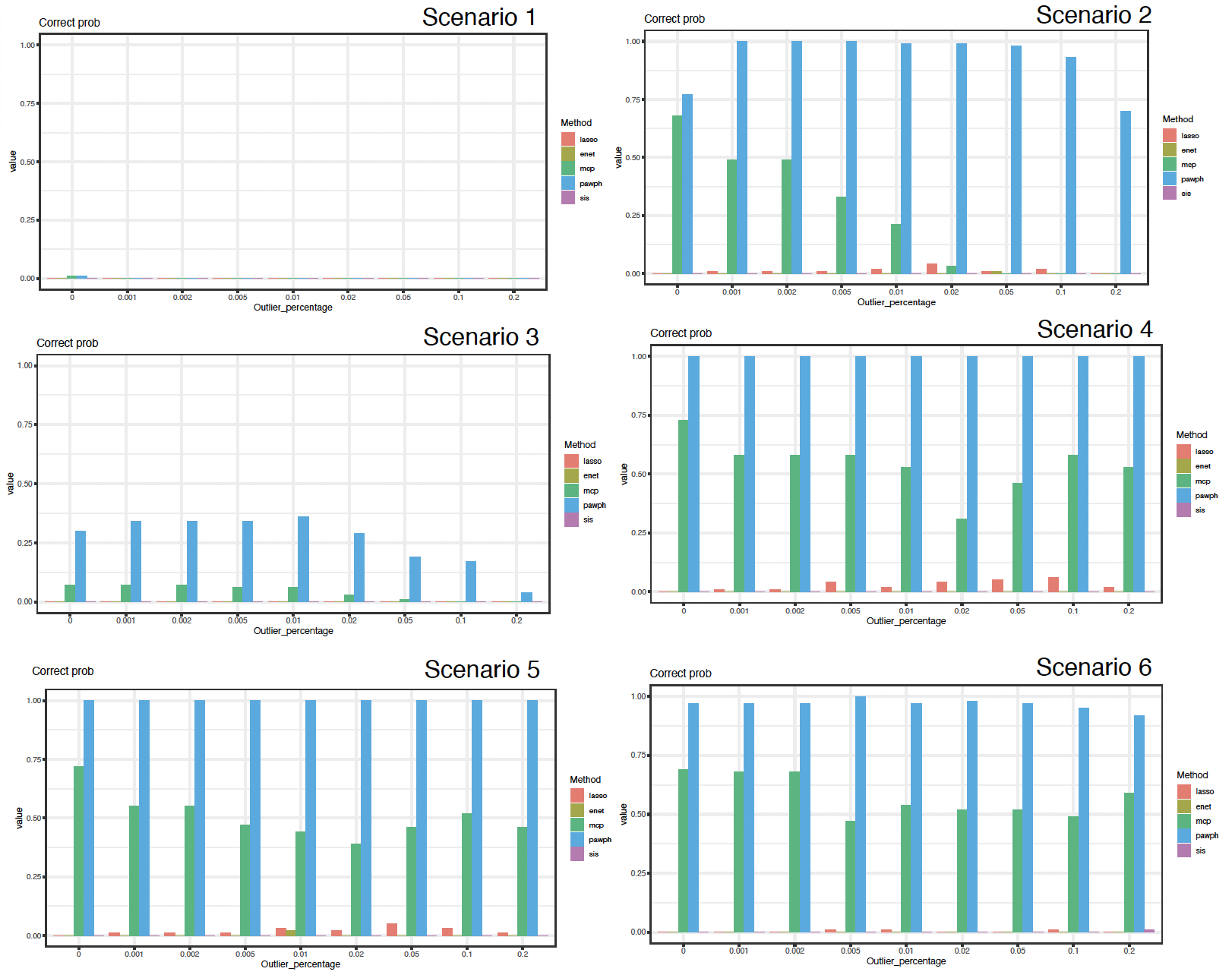


Supplementary Figure 1. Probability of correctly identifying the true model.


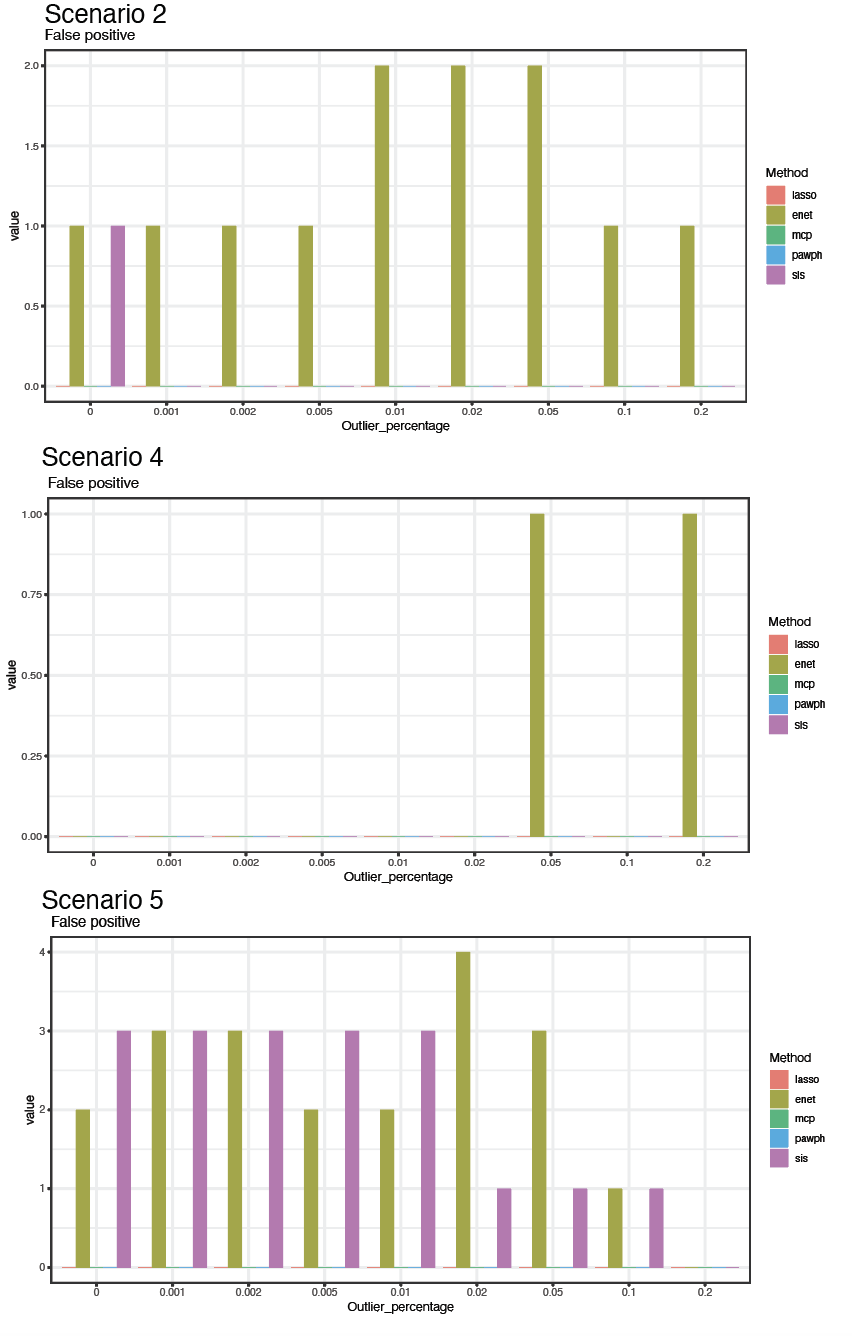


Supplementary Figure 2. Falsely identified variables.


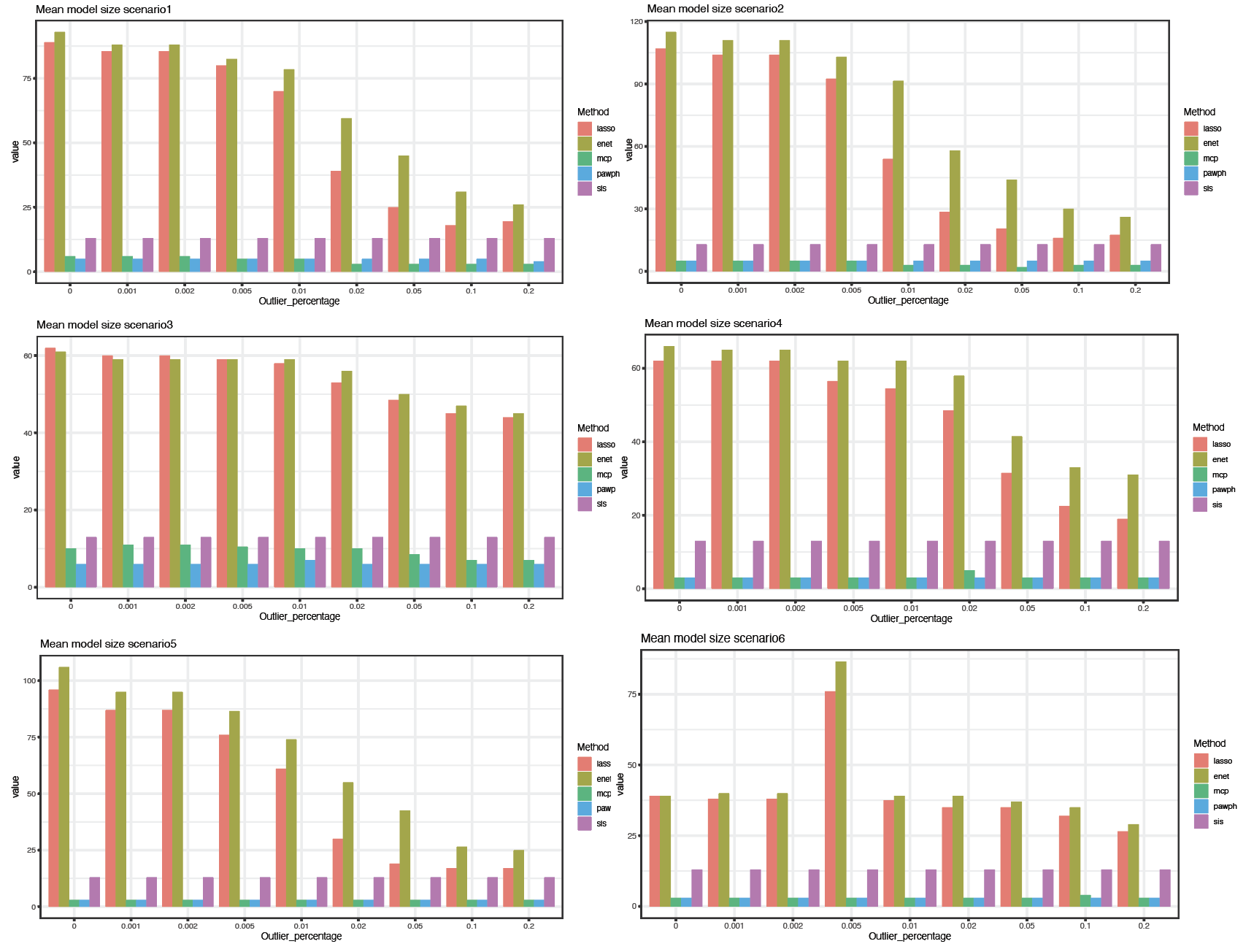


Supplementary Figure 3. Mean model size.

Results:

Real data analysis

We perform a real data analysis on the uveal melanoma cancer survival data [22]. This ultra-high dimensional data contains 80 samples with a censoring rate of 71.25%, each sample has 19533 gene expression measurements. Among all twelve methods, four methods failed to run: namely, the adaptive lasso Cox model, mcp Cox model, scad Cox model, and pawph. The high censoring rate and small sample size present challenges for tuning parameter selection in these methods. Gene CA12, which has been found to be associated with patients’ overall survival outcome in the literature [23,24,25], was identified by both robust methods pawph_mcp and pawph_scad with higher effect estimates compared to those from two non-robust methods, namely lasso Cox and elastic net Cox (Result Figure 1.). Robust methods pawph_mcp, pawph_scad and SIS selected smaller numbers of genes compared to the non-robust lasso Cox and elastic net Cox model. This agrees with our simulation results. Additionally, the SIS method selected different genes compared to other methods, which is expected given the rich correlation structure in the data (Result Figure 2.).

Gene ZBED1, which has also been found in previous studies [23,24,25], was detected by four non-robust methods. However, other identified genes by non-robust methods such as HSD3B2 and BTRC were not found in most of the literature to be related to the uveal melanoma cancer. This may again be due to the high correlation among these genes (Result Figure 2.). By examining the KM curve visually, we observed differences in the expression values for the cancer versus survival group (Result Figure 3.).

We further examine the variable selection performances of these methods when introducing 5% and 20% outliers to this real data, respectively. The variable selection heatmap when introducing 5% outliers shows the same results as compared to the heatmaps when there are no outliers (Result Figure 4). However, differences in the selected variables and their estimates are observed when introducing 20% outliers for both robust and non-robust methods (Result Figure 5).


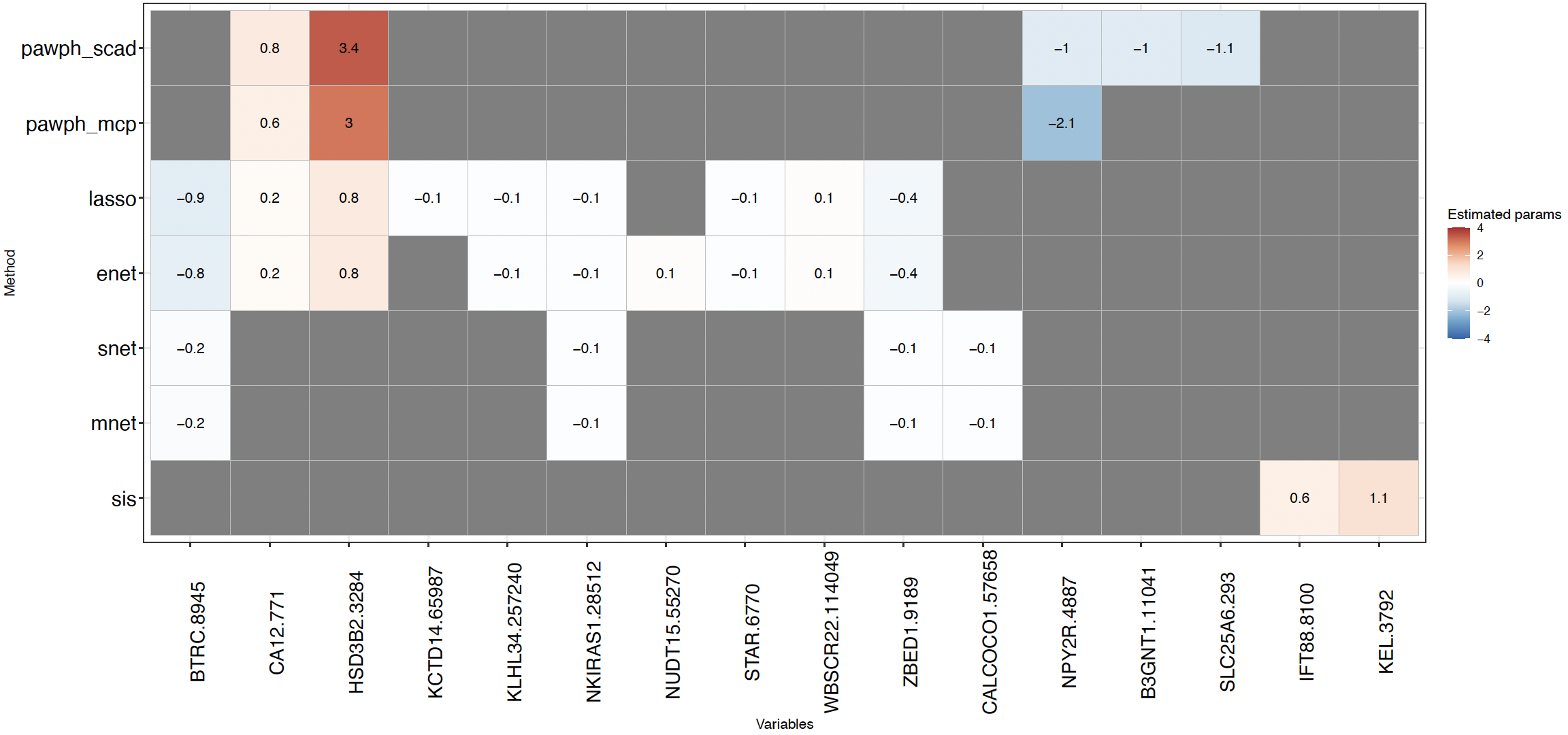


Result Figure 1. Heatmap of selected genes and their estimates.


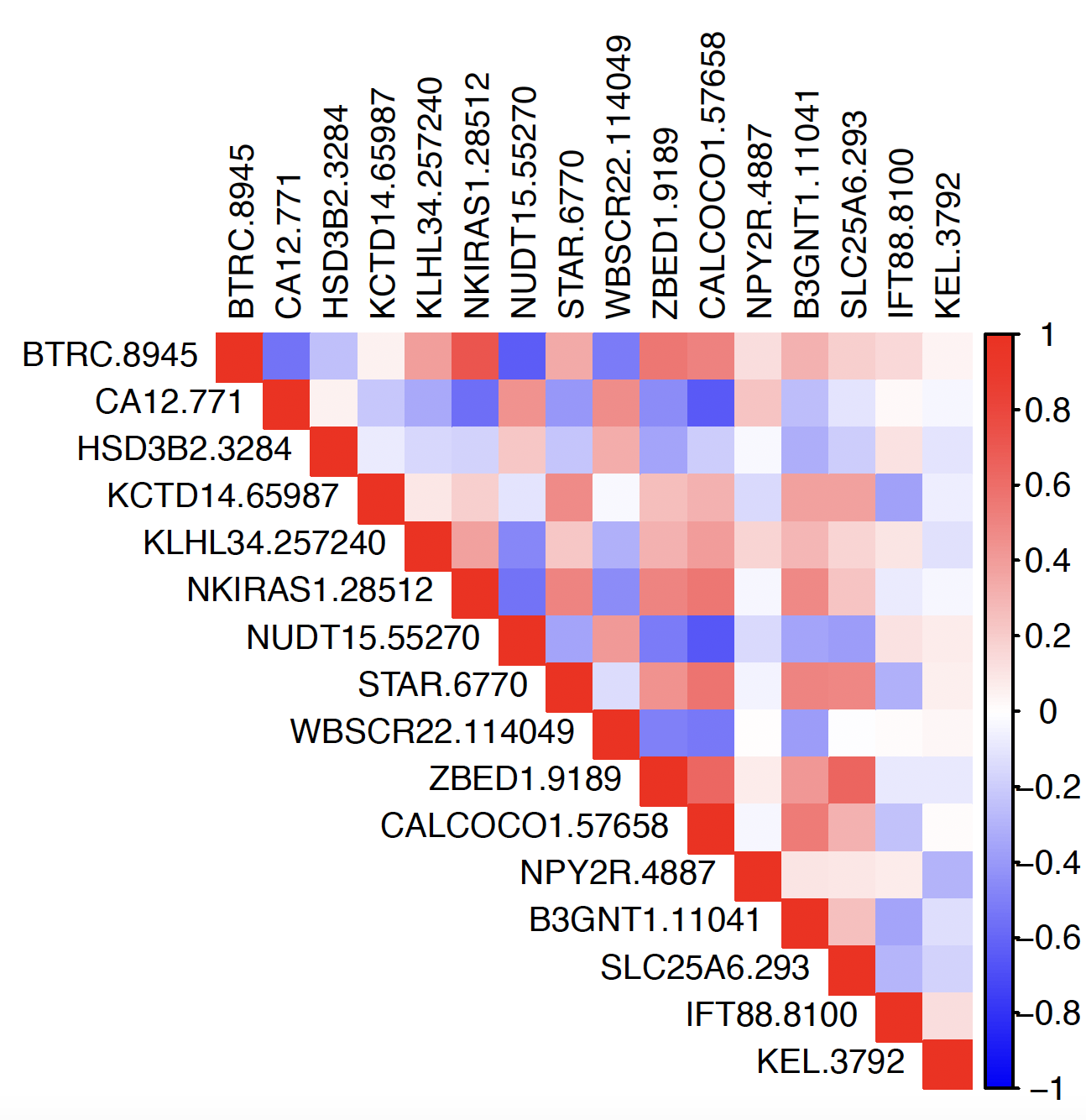


Result Figure 2. Correlation among identified genes.


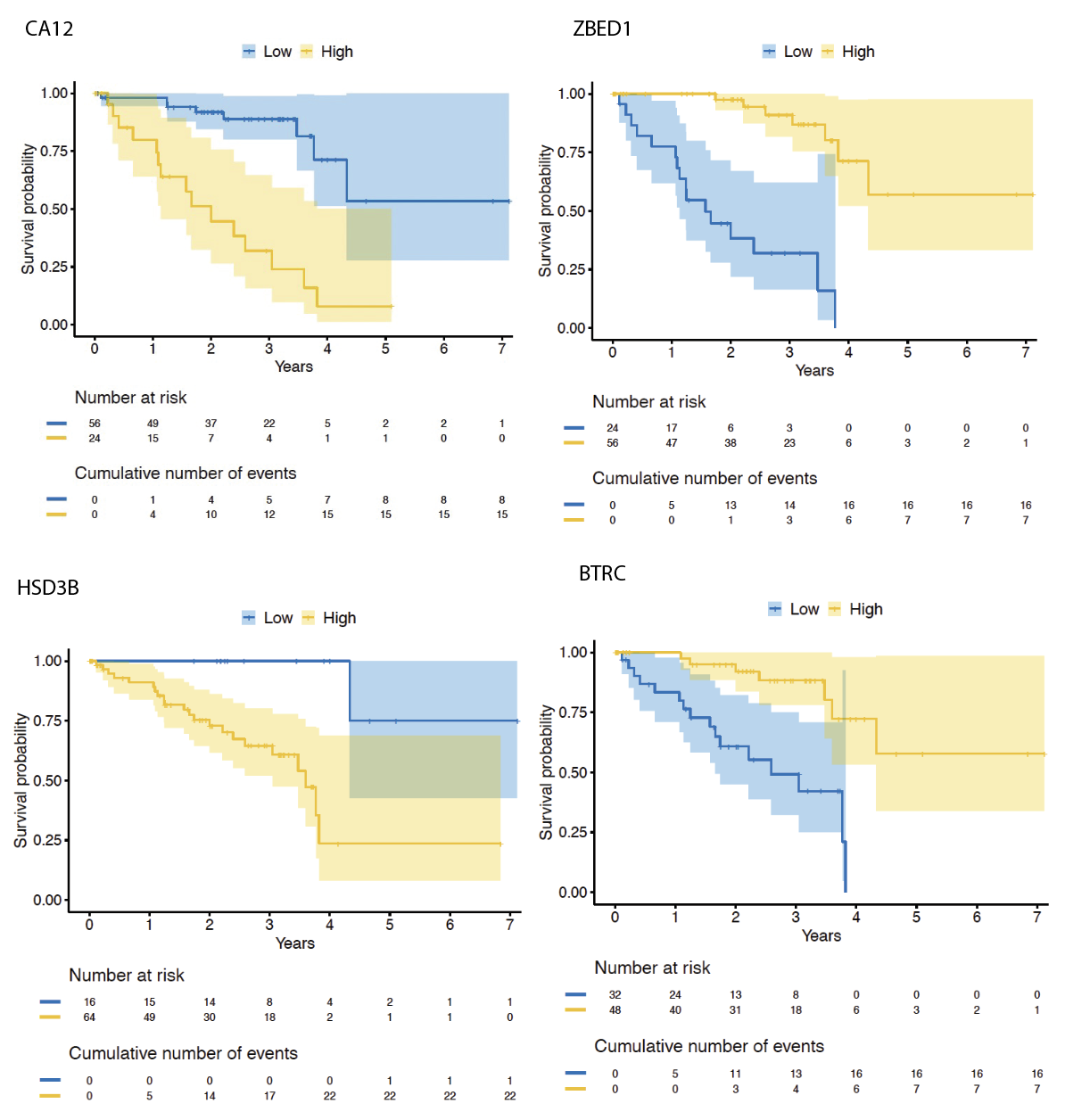


Result Figure 3. Kaplan-Meier curves for example identified genes.


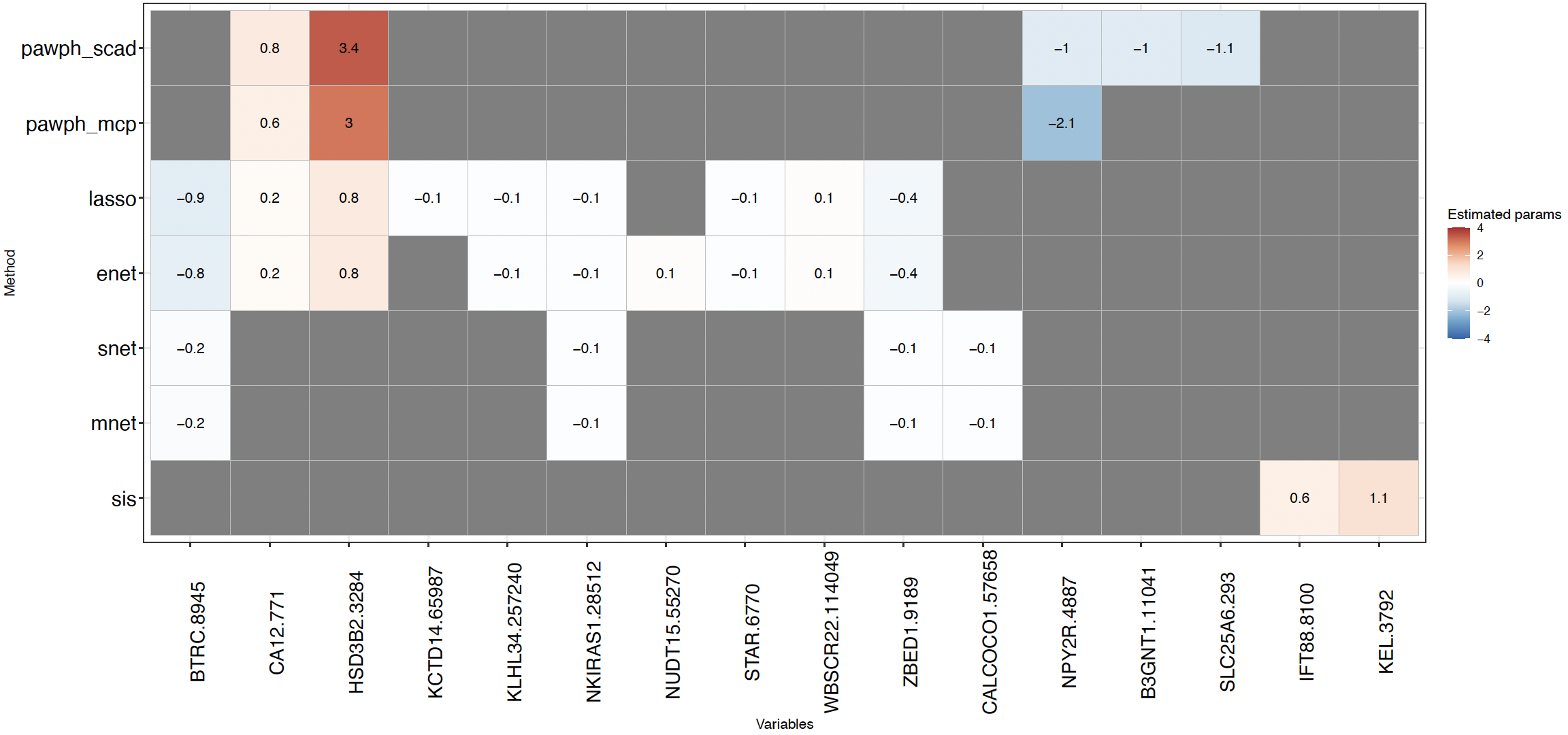


Result Figure 4. Outlier levels 5% results.


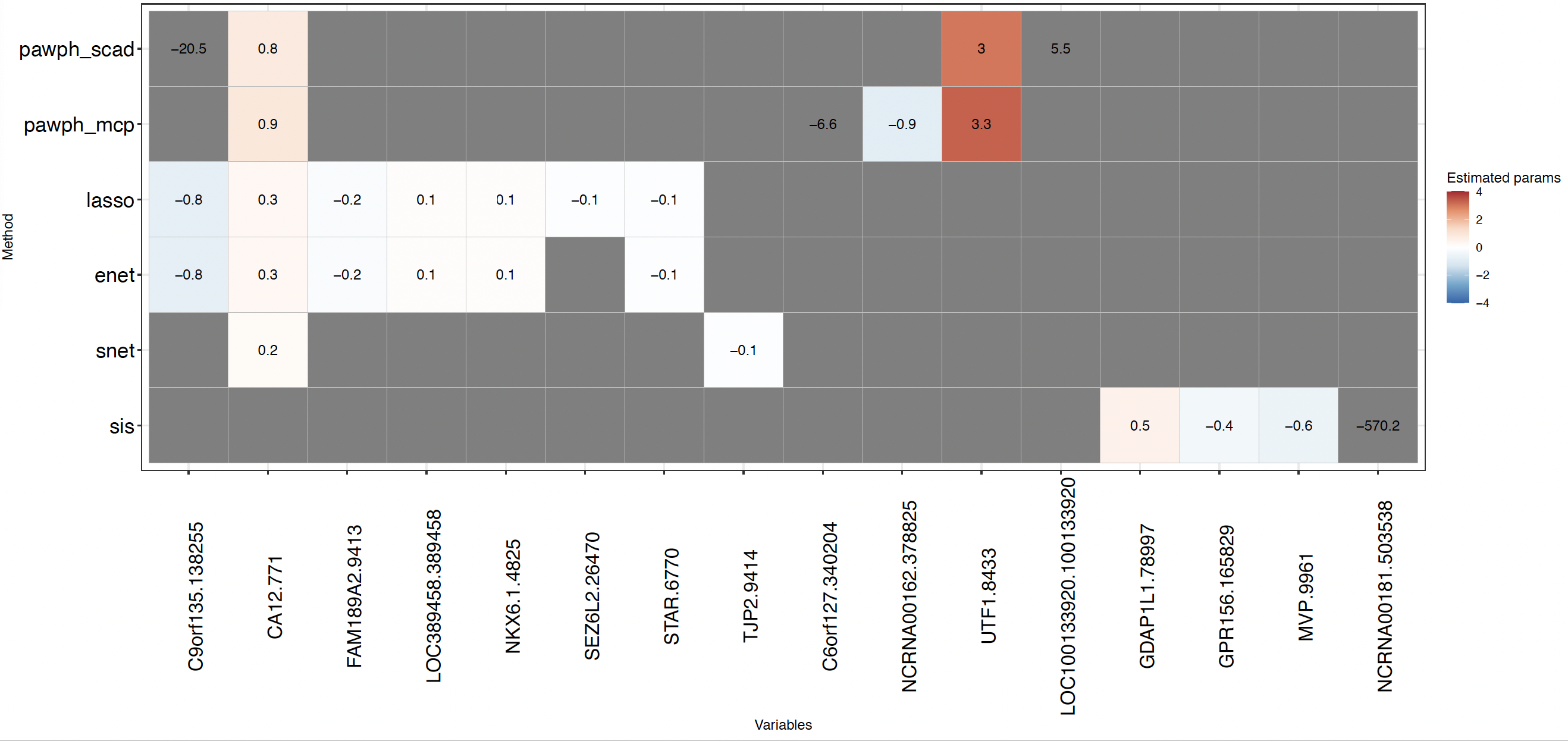


Result Figure 5. Outlier levels 20% results.

References:

[1] Wang, Y., Jiang, Y., Zhang, J., Chen, Z., Xie, B., & Zhao, C. (2022). Robust variable selection based on the random quantile LASSO. *Communications in Statistics-Simulation and Computation*, *51*(1), 29-39.

[2] Wang, H. J., Zhou, J., & Li, Y. (2013). Variable selection for censored quantile regresion. *Statistica Sinica*, *23*(1), 145.

[3] Jiang, Y., Wang, Y., Zhang, J., Xie, B., Liao, J., & Liao, W. (2021). Outlier detection and robust variable selection via the penalized weighted LAD-LASSO method. *Journal of Applied Statistics*, *48*(2), 234-246.
[4] Su, P., Tarr, G., & Muller, S. (2024). Robust variable selection under cellwise contamination. *Journal of Statistical Computation and Simulation*, 1-17.

[5] Bindele, H. F., Denhere, M., & Sun, W. (2022). Generalized signed-rank estimation and selection for the functional linear model. *Statistics*, *56*(4), 719-738.

[6] Xu, J., Leng, C., & Ying, Z. (2010). Rank-based variable selection with censored data. *Statistics and computing*, *20*(2), 165.

[7] Cai, T., Huang, J., & Tian, L. (2009). Regularized estimation for the accelerated failure time model. *Biometrics*, *65*(2), 394-404.

[8] Johnson, B. A. (2009). Rank-based estimation in the ℓ1-regularized partly linear model for censored outcomes with application to integrated analyses of clinical predictors and gene expression data. *Biostatistics*, *10*(4), 659-666.

[9] Liu, Z., Song, Y., & Cheng, Y. (2023). Robust Variable Selection with Exponential Squared Loss for the Spatial Durbin Model. *Entropy*, *25*(2), 249.

[10] Wang, X., Shao, J., Wu, J., & Zhao, Q. (2023). Robust variable selection with exponential squared loss for partially linear spatial autoregressive models. *Annals of the Institute of Statistical Mathematics*, *75*(6), 949-977.

[11] Wang, Y., Wang, Z., & Song, Y. (2023). Robust Variable Selection with Exponential Squared Loss for the Spatial Single-Index Varying-Coefficient Model. *Entropy*, *25*(2), 230.

[12] Zou, H., & Jiang, Y. (2023). Robust variable selection for the varying index coefficient models. *Journal of the Korean Statistical Society*, *52*(4), 767-793.

[13] Wu, Y., Song, Y., Liang, X., & Gai, Y. (2022). Exponential squared loss based robust variable selection of AR models. *Brazilian Journal of Probability and Statistics*, *36*(2), 220-242.

[14] Song, Y., Liang, X., Zhu, Y., & Lin, L. (2021). Robust variable selection with exponential squared loss for the spatial autoregressive model. *Computational statistics & data analysis*, *155*, 107094.

[15] Jiang, Y., Tian, G. L., & Fei, Y. (2019). A robust and efficient estimation method for partially nonlinear models via a new mm algorithm. *Statistical Papers*, *60*(6), 2063-2085.

[16] Kepplinger, D. (2023). Robust variable selection and estimation via adaptive elastic net S-estimators for linear regression. *Computational Statistics & Data Analysis*, *183*, 107730.

[17] Li, B. W., Zhang, Y. Q., & Tang, N. S. (2020). Robust variable selection and estimation in threshold regression model. *Acta Mathematicae Applicatae Sinica, English Series*, *36*(2), 332-346.

[18] Ren, J., Du, Y., Li, S., Ma, S., Jiang, Y., & Wu, C. (2019). Robust network‐based regularization and variable selection for high‐dimensional genomic data in cancer prognosis. *Genetic epidemiology*, *43*(3), 276-291.

[19] Ghosh, A., Jaenada, M., & Pardo, L. (2024). Robust adaptive variable selection in ultra-high dimensional linear regression models. *Journal of Statistical Computation and Simulation*, *94*(3), 571-603.

[20] Shi, X., Liu, J., Huang, J., Zhou, Y., Xie, Y., & Ma, S. (2014). A penalized robust method for identifying gene–environment interactions. *Genetic epidemiology*, *38*(3), 220-230.

[21] Luo, B., Gao, X., & Halabi, S. (2022). Penalized weighted proportional hazards model for robust variable selection and outlier detection. *Statistics in medicine*, *41*(17), 3398-3420.

[22] Cheng, X., Liu, Y., Wang, J., Chen, Y., Robertson, A. G., Zhang, X., Jones, S. J. M., & Taubert, S. (2022). cSurvival: a web resource for biomarker interactions in cancer outcomes and in cell lines. *Briefings in bioinformatics*, *23*(3), bbac090. https://doi.org/10.1093/bib/bbac090

[23] Yin, Y., Du, W., & Li, F. (2022). The construction of a hypoxia-based signature identified CA12 as a risk gene affecting uveal melanoma cell malignant phenotypes and immune checkpoint expression. *Frontiers in oncology*, *12*, 1008770. <https://doi.org/10.3389/fonc.2022.1008770>

[24] Xue, M., Shang, J., Chen, B., Yang, Z., Song, Q., Sun, X., Chen, J., & Yang, J. (2019). Identification of Prognostic Signatures for Predicting the Overall Survival of Uveal Melanoma Patients. *Journal of Cancer*, *10*(20), 4921–4931. <https://doi.org/10.7150/jca.30618>

[25] Lamas, N. J., Martel, A., Nahon-Estève, S., Goffinet, S., Macocco, A., Bertolotto, C., ... & Hofman, P. (2021). Prognostic biomarkers in uveal melanoma: The status quo, recent advances and future directions. *Cancers*, *14*(1), 96.
